# Aroma profiles of shalgam: Effect of purple carrot (*Daucus carota*) amount

**DOI:** 10.1002/fsn3.4164

**Published:** 2024-04-30

**Authors:** Hasan Tanguler, Kemal Sen, Selin Ozge Dinc

**Affiliations:** ^1^ Department of Food Engineering Niğde Ömer Halisdemir University Nigde Turkey; ^2^ Department of Food Engineering Nevsehir Haci Bektas Veli University Nevsehir Turkey; ^3^ Department of Food Technology Canakkale Onsekiz Mart University Canakkale Turkey

**Keywords:** aroma compounds, fermented lactic acid drink, GC‐MS‐FID, purple carrot amount, shalgam

## Abstract

Shalgam is a fermented product characterized by its color and aroma compounds. However, there is no standard regarding the amount of use of purple carrot, which is the major raw material in production and affects the fermentation and aroma compounds of the product. This present research was designed to examine the effect of purple carrot concentrations on aroma compounds, which are one of the most important characteristics that reflect the quality characteristics of shalgam and affect consumer preferences. Aroma compounds in shalgam juices produced using five different amounts of carrots were analyzed by gas chromatography‐flame ionization detector‐mass spectrometry (GC‐FID‐MS). As expected, since the difference between the produced shalgam beverages was only the amount of purple carrots, a qualitative similarity and a quantitative difference were determined in the aroma profiles in general. In the aroma categories defined, terpenes (26 compounds) were the most abundant compounds, followed by esters (17 compounds) and higher alcohols (11 compounds). 88 aroma compounds have been identified in shalgam, and a total of 28 ACs, including 7 terpenes, 7 esters, 3 alcohols, 4 volatile acids, 3 volatile phenols, 1 lactone, 1 norisoprenoid, and 2 naphthalenes, were detected for the first time. The concentration of aromas in the samples varied from 5471.5 to 6490.1 μg/L (*p* < .05). According to principal component analysis, it was determined that the correlation between the position of the shalgam samples in the coordinate system and the aroma groups was significant. This study shows that purple carrots also affect the aroma compounds of shalgam beverages.

## Graphical abstract

Shalgam juice is a nutritious and healthy drink due to the raw materials used in its production (especially black carrots, *Daucus carota*) and fermentation. The main subject of the study is the effect of carrot, which is the basic raw material in the production of shalgam, whose consumption is increasing in some cities of Turkey and Europe, on lactic acid and especially aroma substances, which have an important place in consumer preferences. Quantitatively, the most dominant aromas were determined to be alcohols and terpenes. Additionally, 28 aromas were detected in shalgam for the first time.

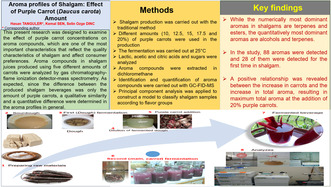

## 1 INTRODUCTION

Shalgam is a traditional soft‐fermented drink. It is an important consumption product with its unique flavor as well as being healthy and nutritious with the high vitamins, minerals, phenolic compounds, and antioxidants it contains. Purple carrot is an important part of the raw materials used in shalgam production due to its rich components such as anthocyanins, phenolics, vitamins, minerals, and lactic acid bacteria (Tanriseven et al., 2020). Its sensory properties mostly consist of the presence of volatile and non‐volatile (free sugars, phosphates, nitrogenous compounds, etc.) compounds from black carrots (Alabran & Mabrouk, 1973; Ekinci et al., 2016; Tanguler et al., 2017). Some researchers have reported that the terpene compounds of black carrots, which have an important place in the aroma compounds of shalgam, usually have a harsh and bitter taste (Ekinci et al., 2016; Tanguler et al., 2017). Similarly, it has been reported that black carrot contributes to flavor development by playing an important role in the conversion of sugar to organic acids during fermentation (Tanguler et al., 2017).

Purple carrot has a total sugar content of 5.12–7.09%, and it contains sucrose (5–33.1 g/100 g), glucose (0.66–5.64 g/100 g), and fructose (0.67–4.36 g/100 g; Erten & Tanguler, 2012). However, it has been reported recently that some producers do not use turnip radish as raw materials by making some modifications in production but only use purple carrots and other raw materials. Purple carrot is an important part of the raw materials utilized in shalgam beverage manufacture (Erten et al., 2008; Tanguler & Erten, 2012).

Aroma is one of the most significant sensorial attributes, reflecting the quality characteristics of shalgam, which is made up of a mixture of volatile molecules with various odor characteristics. Aroma compounds (ACs) are compounds that evaporate easily in ambient conditions. The molecular weight of these compounds is less than 300 Da (Angerosa, 2002). Following changing consumer demands in recent years, the examination of the volatile profiles of the products tends to be one of the primary characteristics to be considered in the progress of fermented food.

Especially the amount of black carrot added is crucial, as it determines the acidity and color levels that determine the sourness of the final beverage. Moreover, the unique aroma properties of shalgam are greatly affected by the raw materials used in its manufacture and the fermentation conditions carried out. Black carrot, in particular, has an important impact on the aroma of shalgam beverage, both because it is a fundamental raw material in the production of shalgam beverage and because of its unique aroma (McFeeters, 2004; Tanguler et al., 2014). Although the number of studies on shalgam beverage, which is stated to have positive effects on health, has increased in recent years, there are a limited number of studies on the flavoring substances in this product. These studies were realized on the effect of the production methods (traditional process, fast process, and usage of starter culture) on the aroma profile of shalgam (Tanguler et al., 2017), the effects of pasteurization and storage conditions on the aroma compounds present (Kirlangic et al., 2021), and the next‐generation sequencing of shalgam beverage aroma influencing microbiota (Ekici et al., 2021). However, no published studies have been found so far on the effect of the amount of black carrot on the composition of the aroma profiles of shalgam. The present research aimed to compare the effects of the amount of purple carrot on the composition of ACs of shalgam. Therefore, in the shalgam produced using different amounts of purple carrots, ACs were determined and quantified by GC–MS and GC‐FID, which are widely used.

## 2 MATERIALS AND METHODS

### 2.1 Materials and chemicals

Black carrots, bulgur flour (setik), and turnip radish used in the production of shalgam beverages were obtained from the Adana fruit and vegetable market. Baker's yeast and salt were obtained from the Migros market. Dichloromethane, anhydrous sodium sulfate, lactic acid, acetic acid, citric acid, glucose, and fructose were bought from Merck KGaA (Darmstadt, Germany).

### 2.2 Shalgam production

**FIGURE 1:**
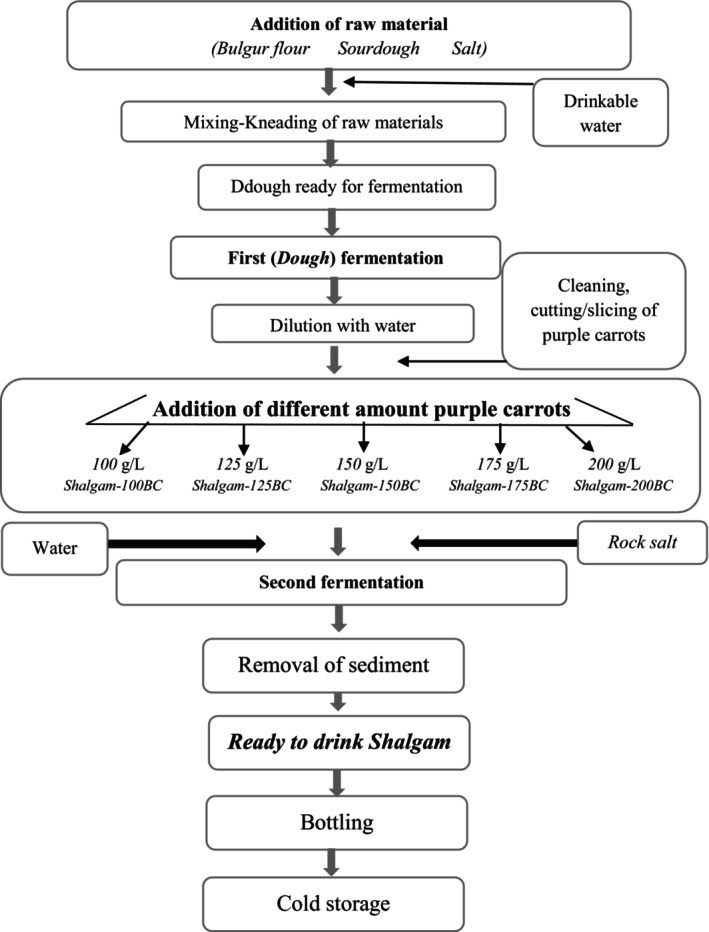
Shalgam production by traditional method. Shalgam‐100PC, 100 g/L purple carrot addition; Shalgam‐125PC, 125 g/L purple carrot addition; Shalgam‐150PC, 150 g/L purple carrot addition; Shalgam‐175PC, 175 g/L purple carrot addition; Shalgam‐200PC, 200 g/L purple carrot addition.

Shalgam beverage production was done with the traditional process with some modifications (Erten et al., 2008). In brief, setik, rock salt, and sourdough were added at rates of 30, 2, and 2 g/L, respectively. Then, drinking water was added and kneaded until it reached dough consistency. The dough was fermented in 40‐L plastic drums at 25°C (Ι. Fermentation). The dough fermentation was carried out for 72 h, and then it was diluted at the end of the process. After this, it was carried out with the main fermentation, also known as carrot fermentation. The first fermentation broth was mixed with different amounts (100, 125, 150, 175, and 200 g/L) of sorted and chopped purple carrots and rock salt (10 g/L). In other words, the fermentation broth was transferred to five separate tanks in equal amounts to carry out carrot fermentation (2nd fermentation). Shalgam tanks were named Shalgam‐100PC, Shalgam‐125PC, Shalgam‐150PC, Shalgam‐175PC, and Shalgam‐200PC, respectively. Glass tanks with a volume of 9 L were used, and the tank was closed before fermentation, which was carried out at 25°C. Sampling is described in Figure 1. No pre‐treatment was applied to purple carrots other than shredding in standard sizes and cleaning. In each fermentation, bulgur flour, rock salt, drinking water, and sourdough were used in the same amount and quality as standard. When the fermentation ended, the shalgam beverages were removed from the residue, bottled, and placed in cold storage at +4°C. They were kept in the same place throughout the analysis. Before the analyses, parallel samples were combined, and analyses were carried out using these samples. Aroma analyses were carried out in three parallels.

### 2.3 HPLC determination of lactic, acetic, and citric acids and sugars

Lactic, acetic, and citric acids and sugars (glucose and fructose) in shalgams were determined by using the HPLC “Shimadzu LC‐20AD, Japan”. The shalgam beverages removed from the residue were firstly filtered (0.45 and 0.22 mm filters; “Millipore, Darmstadt”) and then injected into the column. The column used in the analysis is a “300 × 7.8 mm” long Bio Rad brand (USA) Aminex‐HPX 87H column. The column temperature was 50°C, and high‐purity water and sulfuric acid were used as eluent (0.5% mmol sulfuric acid–water). The flow ratio was set at 0.6 mL per minute, and a UV detector for organic acids and a refractive index detector for sugars were used. The amount of sugar was calculated using an RI detector (Shimadzu). On the other hand, the amounts of organic acids were calculated using a UV detector (Shimadzu; Tanguler & Erten, 2012). Identification of sugar and organic acid peaks was performed using certified standard materials (“Lactic acid Merck‐100366,” “acetic acid Merck‐818755,” and “citric acid Merck‐100244,” and “glucose Merck‐108337” and “fructose Fluka‐47740”). The emergence time and spectra of the peaks were identified by comparison with standard substances. To calculate the concentration of the peaks, five different concentrations of standard substances were injected into the system, and the resulting calibration curves were used for this purpose.

### 2.4 Liquid‐liquid extraction carried out in shalgam samples

The extraction procedure performed in this study was designed in light of our previous article (Tanguler et al., 2017). According to Sen (2021), ACs were extracted in dichloromethane (CH_2_CI_2_). Before extraction, the samples, 4‐nonanol (internal standard) and dichloromethane, were stirred for 30 min at 4°C and their amounts were 40 μg and 40 mL, respectively. The mixing process was carried out under nitrogen gas conditions. After that, centrifugation (9000 ***g***, 15 min) was applied to the obtained mixtures at 4°C. The pooled organic extract was obtained by dehydration with Na_2_SO_4_. The organic extracts were then concentrated to 0.5 mL at 45°C in a “Vigreux column.” Extraction was performed in triplicate for each produced shalgam. The concentrations of ACs are stated as 4‐nonanol equivalent (Sen, 2021). The peak area ratio was adjusted with the response factors of each AC, and they were calculated from the intensity ratio of each compound to 4‐nonanol.

### 2.5 Identification and quantification of aroma compounds with GC‐FID‐MS

The identification and quantification of the ACs were done by using “Agilent 6890” gas chromatography (GC) equipped with a flame ionization detector (FID) and an Agilent 5975‐Network‐mass selective detector (Wilmington, USA). The separations of the ACs were realized using a DB‐WAX capillary column (30 m length × 0.25 mm i.d. × 0.5 mm thickness; J&W Scientific Folsom, USA).

Under these conditions, artifact formation may occur through thermal degradation of the analytes. To reduce this, 3 μL of the extract was injected in a pulsed indivisible (forty psi pressure, 30 s) mode. The set temperature values were 270 and 280°C for the injector and FID detectors, respectively. The DB‐Wax column temperature was programmed to be set as increasing from 50 to 250°C at a rate of 4°C/min, with a final hold at 250°C for 10 min. The same program for temperature was carried out for the mass‐selective detector. The mass spectrometer circumstances were as follows: ionization energy of 70 eV, source temperature of 250°C, quadrupole temperature of 120°C, scan rate of 2.0 scan/s, and mass range of 29–350 mass/charge (m/e). The ACs were identified based on the retention index and reference ACs and mass spectra using a commercial database of spectra (Wiley 7, NIST 98, and Flavor 2L) and the instrument's internal library created from the previous laboratory studies. Some of the compounds were quantified and confirmed by utilizing the internal standard. The retention indexes of the ACs were determined based on an n‐alkane series. Quantifications of the ACs were calculated by the method of internal standards using GC‐FID (Kesen, 2020; Sonmezdag et al., 2018; Türk & Şen, 2021). Identification of ACs was made using GC–MS. The LOQ value varies between 2.4 ng/g and the LOD value varies between 0.92 ng/g in the calculation made on the 4‐nonanol internal standard. As a result, the realized liquid‐liquid extraction method has low LOD and LOQ values.

### 2.6 Statistical analysis

The XLStat (2022) (Addinsoft) package program was used in the statistical analysis. Quantities and standard deviations of the data were found using MS Office Excel. Analysis of variance and Duncan's multiple comparison test methods were used to evaluate differences. Differences of *p* < .05 were considered significant. Principal component analysis (PCA) was also performed. The data matrix consisted of samples and chemical classes of aroma compounds as observations and variables.

## 3 RESULTS AND DISCUSSION

Organic acids, especially lactic acid, found in fermented products are important chemical components that contribute to the taste and aroma of the products (Tanguler & Erten, 2012). During the production of shalgam beverages, especially in the second fermentation, the sugars in the raw materials are mainly converted into lactic acid by mostly homofermentative and also heterofermentative lactic acid bacteria (Erten & Tanguler, 2012). Lactic and acetic acid concentrations in the samples were determined to be between 4.18–6.6 g/L (*p* < .001) and 0.55–0.65 mg/L (*p* > .05), respectively. On the other hand, citric acid could not be detected in any of the shalgam samples. In addition, the amounts of fructose and glucose in the samples were determined between 50.5–57.1 mg/L (*p* > .05) and 42.6–60.5 mg/L (*p* < .05), respectively.

Tanguler and Erten (2012) determined the lactic acid and acetic acid contents of shalgam beverages produced by different methods, collected from the Adana market, between 2.66–4.74 g/L and 0.35–1.16 g/L, respectively. The researchers also could not detect citric acid in two samples but found citric acid between 71.5 and 3417 mg/L in the other samples. In addition, it is reported that in the shalgam standard existing in Turkey, the amount of lactic acid in shalgam beverages should be between 4.5 and 5.5 g/L (TSE, 2003). Ekinci et al. (2016) stated that lactic acid, acetic acid, fructose, and glucose amounts in shalgams were 8.9 g/L, 1.29 g/L, 104 mg/L, and 41 mg/L, respectively. Our results state that the lactic acid and acetic acid levels of shalgam beverages are in good agreement with TSE (2003) and Tanguler and Erten (2012). On the other hand, it was found to be lower than the values reported by Ekinci et al. (2016).

Compared to the raw materials used in shalgam beverage production, the amounts of glucose and fructose in shalgam beverages are generally at low levels. The most important reason for this is the use of these sugars mainly by lactic acid bacteria and also by yeasts during the two‐stage fermentation (Ekinci et al., 2016; Tanguler & Erten, 2012). Tanguler and Erten (2012) stated that glucose and fructose levels in shalgam beverages produced by different methods collected from the Adana market were generally below 200 mg/L and were between 117–1902 mg/L and 60–1310 mg/L, respectively. The glucose and fructose results obtained in this study were generally found to be slightly lower than the results reported by the above‐mentioned researchers.

### 3.1 Aroma compounds of shalgam samples

Although there have been few studies on ACs in shalgams (Kirlangic et al., 2021; Tanguler et al., 2017), no previous study has been found on the effect of adding different amounts of purple carrot. Therefore, this study is the first to focus on this topic, and ACs were determined by GC‐FID‐MS. A total of 88 ACs composed of 26 terpenes, 17 esters, 11 alcohols, 10 volatile acids, 9 volatile phenols, 6 lactones, 2 norisoprenoids, 5 naphthalenes, and 2 aldehydes and ketone were identified in the shalgam samples (Table 1). Terpenes were the most abundant of the identified categories of ACs, followed by esters.

**TABLE 1:** Aroma compounds of shalgams (μg/L).

	LRI	Shalgam‐100PC	Shalgam‐125PC	Shalgam‐150PC	Shalgam‐175PC	Shalgam‐200PC	ID
Terpene compounds							
α‐Pinene	1013	(36.78 ± 1.12)^b^	(40.82 ± 1.82)^b^	(45.42 ± 1.89)^ab^	(38.66 ± 1.34)^b^	(59.29 ± 2.15)^a^	LRI, MS, Std
β‐Pinene	1108	(39.23 ± 1.84)^a^	(20.80 ± 0.74)^b^	(12.26 ± 0.42)^c^	(21.37 ± 0.93)^b^	(33.75 ± 1.48)^a^	LRI, MS, Tent
β‐Myrcene	1145	(15.41 ± 0.64)^b^	(10.11 ± 0.37)^c^	(12.68 ± 0.57)^c^	(14.51 ± 0.61)^b^	(23.84 ± 1.07)^a^	LRI, MS, Std
DL‐Limonene	1213	(4.66 ± 0.15) ^a^	(4.97 ± 0.18)^a^	(5.16 ± 0.23)^a^	(4.75 ± 0.20)^a^	(4.21 ± 0.16)^b^	LRI, MS, Std
γ‐Terpinene	1220	(24.06 ± 0.96)^b^	(29.46 ± 1.21)^a^	(22.58 ± 0.87)^b^	(23.28 ± 0.63)^b^	(27.42 ± 0.52)^a^	LRI, MS, Std
α‐Terpinolene	1232	(390.96 ± 9.15)^ab^	(368.14 ± 12.24)^b^	(409.22 ± 12.37)^a^	(418.26 ± 10.26)^a^	(428.38 ± 15.63)^a^	LRI, MS, Std
(Z)‐Linalool oxide	1424	(27.52 ± 1.22)^c^	(28.97 ± 1.33)^c^	(31.13 ± 1.32)^b^	(34.84 ± 1.52)^ab^	(36.14 ± 1.19)^a^	LRI, MS, Std
(E)‐Linalool oxide	1453	(66.82 ± 2.50)^c^	(68.24 ± 2.28)^c^	(73.43 ± 2.28)^b^	(87.51 ± 2.96)^a^	(92.55 ± 3.81)^a^	LRI, MS, Std
Vitispirane	1507	(9.09 ± 0.27)^a^	(3.67 ± 0.11)^b^	(2.98 ± 1.32)^c^	(2.85 ± 0.10)^c^	(3.15 ± 0.09)^bc^	LRI, MS, Std
Linalool	1536	(19.76 ± 0.78)^c^	(11.96 ± 0.36)^d^	(24.26 ± 1.18)^b^	(22.18 ± 1.07)^b^	(33.09 ± 1.42)^a^	LRI, MS, Std
4‐Terpineol	1581	(4.85 ± 0.18)^b^	(4.15 ± 0.18)^c^	(4.93 ± 0.22)^b^	(5.18 ± 0.23)^ab^	(5.67 ± 0.12)^a^	LRI, MS, Std
(E)‐Caryophyllene*	1586	(5.27 ± 0.20)^c^	(6.14 ± 0.22)^b^	(6.77 ± 0.14)^b^	(7.12 ± 0.27)^a^	(7.24 ± 0.16)^a^	LRI, MS, Std
(E)‐Pinocarveol	1626	(8.26 ± 0.32)^c^	(8.95 ± 0.20)^bc^	(9.74 ± 0.30)^b^	(11.39 ± 0.34)^a^	(12.99 ± 0.47)^a^	LRI, MS, Std
α‐ Caryophyllene	1656	(8.22 ± 0.24)^c^	(9.67 ± 0.41)^b^	(7.61 ± 0.25)^cd^	(13.20 ± 0.41)^a^	(4.34 ± 0.34)^d^	LRI, MS, Std
Lavandulol	1659	(1.98 ± 0.07)^c^	(2.64 ± 0.11)^ab^	(2.42 ± 0.08)^b^	(3.13 ± 0.13)^a^	(2.99 ± 0.11) ^a^	LRI, MS, Std
1,8‐Menthadien‐4‐ol	1663	(17.05 ± 0.73)^a^	(16.75 ± 0.73)^b^	(17.37 ± 0.61)^a^	(16.53 ± 0.47)^b^	(18.08 ± 0.39)^a^	LRI, MS, Tent
Trans‐β‐farnesene*	1667	(2.37 ± 0.09)^c^	(3.42 ± 0.13)^b^	(2.93 ± 0.12)^bc^	(3.87 ± 0.15)^a^	(2.14 ± 0.07)^c^	LRI, MS, Tent
1‐Borneol	1677	(123.82 ± 4.88)^a^	(96.43 ± 4.31)^b^	(83.65 ± 3.61)^c^	(61.21 ± 2.40)^d^	(66.24 ± 1.96)^d^	LRI, MS, Std
α‐Zingiberene*	1718	(8.86 ± 0.36)^ab^	(9.36 ± 0.28)^a^	(7.68 ± 0.23)^c^	(8.23 ± 0.31)^b^	(6.91 ± 0.29)^d^	LRI, MS, Std
(Z)‐γ‐Bisabolene	1725	(11.16 ± 0.44)^c^	(18.24 ± 0.49)^b^	(17.02 ± 0.52)^b^	(22.33 ± 1.01)^a^	(20.81 ± 0.67)^a^	LRI, MS, Std
(E)‐γ‐Bisabolene	1753	(142.44 ± 5.52)^a^	(38.64 ± 1.37)^d^	(49.78 ± 4.11)^c^	(141.03 ± 5.23)^a^	(96.51 ± 3.94)^b^	LRI, MS, Std
Myrtenol	1769	(28.53 ± 1.33)^b^	(20.19 ± 0.77)^c^	(32.91 ± 1.24)^b^	(30.45 ± 1.29)^b^	(41.41 ± 1.38)^a^	LRI, MS, Std
Nerol*	1795	(47.29 ± 1.89)^a^	(39.97 ± 1.56)^b^	(35.11 ± 1.16)^bc^	(30.47 ± 1.44)^c^	(27.37 ± 1.02)^d^	LRI, MS, Std
Trans‐carveol*	1811	(19.23 ± 0.75)^b^	(38.36 ± 1.23)^a^	(10.88 ± 0.41)^d^	(18.81 ± 0.75)^b^	(13.37 ± 0.24)^c^	LRI, MS, Std
Geraniol	1832	(21.81 ± 0.67)^d^	(26.69 ± 1.18)^c^	(29.20 ± 1.20)^b^	(31.74 ± 1.06) ^b^	(41.64 ± 1.62)^a^	LRI, MS, Std
Squalene*	3103	(106.05 ± 3.64)^a^	(78.93 ± 2.91)^cd^	(91.22 ± 2.87)^b^	(70.11 ± 2.61) ^d^	(84.52 ± 3.32)^c^	LRI, MS, Tent
Esters							
Butyl acetate	1064	(7.75 ± 0.26)^a^	(2.01 ± 0.08)^c^	(1.71 ± 0.03)^d^	(2.75 ± 0.06)^b^	(1.13 ± 0.01)^e^	LRI, MS, Std
Isoamyl acetate	1118	(39.47 ± 1.55)^a^	(20.03 ± 0.72)^c^	(23.99 ± 1.02)^bc^	(17.82 ± 0.44)^d^	(25.18 ± 1.07)^b^	LRI, MS, Std
Ethyl lactate*	1296	(43.57 ± 1.99)^a^	(36.24 ± 1.23)^b^	(41.14 ± 1.61)^a^	(39.33 ± 1.33)^ab^	(34.86 ± 1.44)^b^	LRI, MS, Std
Bornyl acetate	1561	(2.96 ± 0.12)^d^	(4.48 ± 0.19)^b^	(3.15 ± 0.10)^c^	(5.63 ± 0.11)^ab^	(6.32 ± 0.26)^a^	LRI, MS, Std
Ethyl decanoate	1640	(13.90 ± 0.51)^a^	(9.24 ± 0.34)^c^	(12.87 ± 0.39)^ab^	(11.39 ± 0.40)^b^	(13.42 ± 0.52)^a^	LRI, MS, Std
Diethyl succinate	1650	(10.55 ± 0.35)^a^	(7.83 ± 0.26)^b^	(6.41 ± 0.28)^c^	(5.04 ± 0.17)^d^	(6.99 ± 0.31)^bc^	LRI, MS, Std
Dimethyl glutarate*	1669	(82.64 ± 3.21)^a^	(81.73 ± 2.96)^a^	(84.27 ± 3.64)^a^	(79.97 ± 3.55)^a^	(80.33 ± 2.96)^a^	LRI, MS, Tent
Methyl salicylate	1727	(3.91 ± 0.07)^a^	(2.80 ± 0.04)^b^	(2.35 ± 0.08)^c^	(1.28 ± 0.02)^d^	(1.35 ± 0.03)^d^	LRI, MS, Std
2‐Phenylethyl acetate	1778	(10.17 ± 0.28)^d^	(13.29 ± 0.46)^b^	(12.88 ± 0.33)^b^	(11.54 ± 0.41)^c^	(14.04 ± 0.62)^a^	LRI, MS, Tent
Ethyl hexadecanoate	2268	(54.14 ± 2.11)^a^	(29.02 ± 1.24)^d^	(39.68 ± 1.77)^c^	(25.72 ± 1.18)^e^	(49.55 ± 2.16)^b^	LRI, MS, Std
Ethyl‐9‐hexadecanoate	2293	(10.87 ± 0.19)^c^	(12.56 ± 0.37)^a^	(10.81 ± 0.29)^c^	(11.75 ± 0.47)^b^	(9.49 ± 0.40)^d^	LRI, MS, Std
Ethyl octadecanoate*	2488	(22.88 ± 0.84)^b^	(22.07 ± 0.96)^b^	(28.74 ± 1.27)^a^	(19.19 ± 0.81)^c^	(17.93 ± 0.36)^d^	LRI, MS, Std
Ethyl oleate*	2497	(37.12 ± 1.65)^e^	(44.33 ± 1.84)^d^	(51.42 ± 2.48)^c^	(72.73 ± 3.32)^b^	(86.14 ± 4.11)^a^	LRI, MS, Std
Ethyl linoleate*	2544	(29.74 ± 1.12)^a^	(19.59 ± 0.79)^b^	(8.30 ± 0.37)^d^	(12.26 ± 0.57)^c^	(18.72 ± 0.83)^b^	LRI, MS, Std
Ethyl vanillate*	2585	(14.56 ± 0.53)^b^	(26.42 ± 1.03)^a^	(5.23 ± 0.16)^e^	(12.53 ± 0.38)^d^	(11.20 ± 0.27)^c^	LRI, MS, Std
Benzyl benzoate	2594	(11.88 ± 0.34)^c^	(14.55 ± 0.55)^b^	(10.28 ± 0.31)^d^	(20.34 ± 0.73)^a^	(19.17 ± 0.64)^a^	LRI, MS, Std
Ethyl linolenate*	2602	(23.40 ± 0.87)^b^	(27.51 ± 1.10) ^a^	(26.65 ± 0.55)^a^	(23.92 ± 1.02)^b^	(21.20 ± 0.88)^c^	LRI, MS, Std
Higher alcohols							
2‐Butanol	994	(8.88 ± 0.82)^b^	(10.28 ± 0.26)^a^	(7.59 ± 0.19)^c^	(7.48 ± 0.22)^c^	(9.27 ± 0.41)^ab^	LRI, MS, Std
1‐Propanol	1036	(22.04 ± 0.94)^d^	(25.67 ± 1.16)^c^	(26.74 ± 1.12)^c^	(30.58 ± 1.38)^b^	(39.49 ± 1.60)^a^	LRI, MS, Std
2‐Methyl‐1‐propanol	1090	(15.21 ± 0.43)^c^	(16.12 ± 0.67)^b^	(18.06 ± 0.79)^a^	(16.13 ± 0.65)^b^	(14.88 ± 0.59)^c^	LRI, MS, Std
3‐ Methyl‐1‐propanol*	1099	(23.53 ± 1.12)^bc^	(24.82 ± 0.98)^b^	(22.93 ± 0.92)^c^	(18.75 ± 0.77)^d^	(28.03 ± 1.28)^a^	LRI, MS, Std
1‐Butanol	1137	(32.51 ± 1.24)^a^	(34.41 ± 1.23)^a^	(30.81 ± 1.17)^ab^	(27.43 ± 1.21)^b^	(21.21 ± 0.89)^c^	LRI, MS, Std
Isoamyl alcohol	1209	(1677.48 ± 54.48) ^c^	(1555.36 ± 33.17)^c^	(1483.49 ± 28.79)^d^	(1838.69 ± 38.44)^b^	(2206.29 ± 41.83)^a^	LRI, MS, Std
DL‐6‐Methyl‐5‐hepten‐2‐ol	1449	(90.01 ± 3.60)^a^	(81.74 ± 3.23)^b^	(80.74 ± 2.71)^b^	(86.45 ± 4.22)^b^	(71.17 ± 2.16)^c^	LRI, MS, Tent
1‐Octanol*	1550	(14.58 ± 0.37)^a^	(6.61 ± 0.28)^d^	(14.05 ± 0.44)^a^	(10.88 ± 0.38)^c^	(13.59 ± 0.30)^b^	LRI, MS, Std
Benzyl alcohol*	1835	(460.71 ± 18.17)^a^	(430.86 ± 21.11)^ab^	(422.22 ± 11.16)^b^	(409.85 ± 16.73)^b^	(398.77 ± 13.87)^b^	LRI, MS, Std
2‐Phenyl ethanol	1868	(314.77 ± 11.25)^d^	(412.77 ± 18.14)^c^	(635.85 ± 22.97)^b^	(492.59 ± 17.81)^c^	(746.50 ± 19.46)^a^	LRI, MS, Std
2‐Methoxy‐phenyl‐ethanol	1989	(361.15 ± 9.74)^a^	(351.13 ± 14.63)^a^	(344.78 ± 9.26)^a^	(282.25 ± 10.58)^b^	(315.95 ± 8.77)^a^	LRI, MS, Std
Volatile acids							
Acetic acid	1409	(37.75 ± 1.29)^a^	(27.14 ± 1.27)^b^	(17.14 ± 0.56)^c^	(15.69 ± 0.41)^d^	(14.41 ± 0.29)^e^	LRI, MS, Std
Butanoic acid	1597	(6.44 ± 0.22)^b^	(5.33 ± 0.19)^c^	(7.57 ± 0.18)^b^	(21.29 ± 1.02)^a^	(0.84 ± 0.01)^d^	LRI, MS, Std
Isovaleric acid	1638	(3.28 ± 0.11)^b^	(3.69 ± 0.14)^a^	(3.48 ± 0.06)^ab^	(2.60 ± 0.09)^c^	(3.03 ± 0.10)^b^	LRI, MS, Std
2‐methyl‐butanoic acid*	1640	(19.04 ± 0.86)^a^	(15.76 ± 0.31)^d^	(19.98 ± 0.64)^a^	(18.14 ± 0.71)^b^	(16.49 ± 0.72)^c^	LRI, MS, Tent
Pentanoic acid	1705	(4.17 ± 0.14)^a^	(3.25 ± 0.12)^b^	(1.55 ± 0.03)^d^	(2.76 ± 0.05)^c^	(1.43 ± 0.04)^d^	LRI, MS, Std
Hexanoic acid	1817	(88.46 ± 1.18)^a^	(75.67 ± 2.36)^b^	(92.61 ± 3.81)^a^	(69.75 ± 2.80)^c^	(63.63 ± 3.09)^c^	LRI, MS, Std
Heptanoic acid	1923	(153.08 ± 4.48)^a^	(117.75 ± 2.34)^c^	(136.82 ± 4.89)^b^	(123.10 ± 3.77)^c^	(142.77 ± 5.48)^b^	LRI, MS, Std
Octanoic acid*	2035	(8.65 ± 0.38)^a^	(6.21 ± 0.24)^c^	(9.46 ± 0.41)^a^	(7.68 ± 0.32)^b^	(7.28 ± 0.26)^b^	LRI, MS, Std
Decanoic acid*	2252	(48.23 ± 1.82)^b^	(62.80 ± 2.54)^a^	(37.72 ± 1.20)^c^	(22.05 ± 1.01)^d^	(15.20 ± 0.57)^e^	LRI, MS, Std
Dodecanoic acid*	2476	(6.19 ± 0.24)^b^	(7.41 ± 0.32)^a^	(5.87 ± 0.16)^c^	(4.10 ± 0.14)^d^	(4.25 ± 0.16)^d^	LRI, MS, Std
Volatile phenols							
3‐Methylacetophenone	1731	(5.91 ± 0.21)^d^	(9.53 ± 0.37)^a^	(7.24 ± 0.29)^c^	(8.65 ± 0.31)^b^	(6.23 ± 0.18)^d^	LRI, MS, Tent
Guaiacol*	1813	(27.89 ± 1.28)^d^	(39.89 ± 1.74)^b^	(58.64 ± 1.77)^a^	(34.31 ± 1.35)^c^	(22.55 ± 1.06)^e^	LRI, MS, Std
4‐methyl‐2,6‐di‐tert‐butyl‐phenol	1899	(52.57 ± 2.26)^c^	(89.01 ± 3.98)^a^	(60.18 ± 2.22)^b^	(51.44 ± 1.64)^c^	(53.47 ± 1.58)^c^	LRI, MS, Tent
2‐methoxy‐4‐vinyl‐phenol*	1912	(23.08 ± 1.13)^a^	(nd)^e^	(14.69 ± 0.32)^c^	(16.21 ± 0.70)^b^	(10.78 ± 0.40)^d^	LRI, MS, Tent
Eugenol	2124	(56.03 ± 1.65)^a^	(53.40 ± 1.86)^a^	(31.77 ± 1.08)^c^	(35.64 ± 1.11)^b^	(38.57 ± 1.22)^b^	LRI, MS, Std
p‐Vinyl guaiacol	2146	(15.44 ± 0.42)^b^	(11.76 ± 0.32)^c^	(12.37 ± 0.52)^c^	(17.50 ± 0.66)^a^	(15.34 ± 0.43)^b^	LRI, MS, Std
cis‐isoeugenol*	2299	(12.66 ± 0.49)^b^	(23.98 ± 1.12)^a^	(10.28 ± 0.31)^c^	(12.41 ± 0.29)^b^	(12.28 ± 0.51)^b^	LRI, MS, Std
Acetovanillone	2582	(6.88 ± 0.15)^b^	(7.01 ± 0.31)^b^	(7.18 ± 0.22)^b^	(7.23 ± 0.26)^b^	(11.93 ± 0.47)^a^	LRI, MS, Std
Propiovanillone	2644	(6.50 ± 0.11)^e^	(28.69 ± 1.22)^a^	(9.13 ± 0.40)^c^	(7.26 ± 0.33)^d^	(13.01 ± 0.50)^b^	LRI, MS, Std
Lactones							
γ‐Butyrolactone	1565	(5.64 ± 0.18)^a^	(1.83 ± 0.74)^e^	(4.96 ± 1.99)^b^	(3.32 ± 0.14)^c^	(2.09 ± 0.08)^d^	LRI, MS, Std
γ‐Hexalactone	1647	(2.12 ± 0.08)^c^	(3.60 ± 1.38)^a^	(2.59 ± 0.10)^bc^	(1.57 ± 0.06)^d^	(2.93 ± 0.12)^b^	LRI, MS, Std
γ‐Nonalactone	1992	(53.80 ± 1.95)^c^	(51.96 ± 2.34)^c^	(66.19 ± 2.7)^b^	(73.05 ± 3.01)^a^	(39.32 ± 1.81)^d^	LRI, MS, Std
δ‐Dodecalactone	2414	(21.37 ± 0.96)^bc^	(20.31 ± 0.82)^c^	(7.09 ± 0.22)^d^	(37.71 ± 1.47)^a^	(23.79 ± 1.01)^b^	LRI, MS, Std
Massoilactone	2446	(10.26 ± 0.41)^d^	(13.73 ± 0.42) ^c^	(16.16 ± 0.66)^b^	(19.55 ± 0.70)^a^	(13.43 ± 0.42)^c^	LRI, MS, Tent
Dihydro‐4‐hydoksi‐2(3H)‐furanone*	2527	(6.45 ± 0.21)^c^	(7.91 ± 0.34)^b^	(4.94 ± 0.17) ^d^	(8.43 ± 0.33)^b^	(10.65 ± 0.40)^a^	LRI, MS, Tent
Norisoprenoids							
3‐Hydroxy‐β‐damascenone	2498	(47.54 ± 2.15)^b^	(53.43 ± 1.95)^a^	(55.13 ± 2.11)^a^	(54.29 ± 2.26)^a^	(51.77 ± 2.04)^a^	LRI, MS, Tent
3‐oxo‐α‐ionol*	2604	(8.56 ± 0.36)^a^	(5.11 ± 0.21)^b^	(8.89 ± 0.42)^a^	(8.71 ± 0.34)^a^	(4.42 ± 0.16)^c^	LRI, MS, Tent
Naphthalenes							
Naphthalene	1685	(200.99 ± 8.82)^a^	(169.90 ± 4.16)^b^	(163.79 ± 6.27)^b^	(187.88 ± 5.55)^ab^	(164.88 ± 6.11)^b^	LRI, MS, Std
1‐Methyl‐naphthalene*	1805	(5.11 ± 0.22)^b^	(2.13 ± 0.09)^d^	(7.63 ± 0.25)^a^	(1.96 ± 0.06)^e^	(4.94 ± 0.13)^c^	LRI, MS, Std
2‐Methyl‐naphthalene	1842	(1.64 ± 0.07)^c^	(3.82 ± 0.07)^b^	(3.61 ± 0.14)^b^	(3.51 ± 0.12)^b^	(4.52 ± 0.16)^a^	LRI, MS, Std
1‐Ethyl‐naphthalene*	1904	(20.02 ± 0.81)^b^	(19.17 ± 0.64)^b^	(46.11 ± 2.09)^a^	(49.55 ± 2.22)^a^	(51.13 ± 2.32)^a^	LRI, MS, Std
2‐Ethyl‐naphthalene	1915	(137.16 ± 4.78)^b^	(149.97 ± 3.71)^a^	(146.28 ± 6.37)^a^	(143.31 ± 5.59)^a^	(136.64 ± 4.88)^b^	LRI, MS, Std
Aldehydes and ketones							
Heptanal	1182	(1.21 ± 0.04)^b^	(1.32 ± 0.04)^ab^	(1.28 ± 0.02)^b^	(1.48 ± 0.02)^a^	(1.14 ± 0.04)^c^	LRI, MS, Std
4‐Hydroxy‐4‐methyl‐2‐pentanone	1310	(45.52 ± 1.84)^c^	(49.87 ± 2.38)^b^	(52.05 ± 2.05)^b^	(61.06 ± 2.36)^a^	(48.73 ± 2.11)^b^	LRI, MS, Std

*Note*: Linear retention index values were calculated on the DB‐WAX capillary column; the values are the means of three repetitions as μg/L; The standard deviation of all ACs was <5%; the differences between the amounts shown by different letters on the same line are statistically important (*p* < .05).

Abbreviations: ID, methods of identification; LRI, linear retention index; MS tent, tentatively identified by mass spectrometry; Nd, not detected; Shalgam‐100PC, 100 g/L purple carrot addition; Shalgam‐125PC, 125 g/L purple carrot addition; Shalgam‐150PC, 150 g/L purple carrot addition; Shalgam‐175PC, 175 g/L purple carrot addition; Shalgam‐200PC, 200 g/L purple carrot addition; Std, chemical standard.

* Refers to the ACs found for the first time in shalgams.

The dominant aroma compounds in terms of quantity were higher alcohols (2950 and 3865 μg/L), followed by terpenes (1006 and 1194 μg/L). Tanguler et al. (2017), Ekici et al. (2021), and Kirlangic et al. (2021) defined terpenes, alcohols, ketones, and aldehydes as ACs in shalgam and stated that the main groups of ACs quantitatively were terpenes and alcohols. Tanguler et al. (2017) determined the effect of starter culture addition on ACs with traditional and rapid methods by the GC–MS‐FID method. Ekici et al. (2021) used the SPME/GC–MS method to determine the effect of natural microflora on aroma compounds during 20‐day shalgam fermentation. In another study, the effect of pasteurization and storage on the aroma profile was determined (Kirlangic et al., 2021). These researchers stated that they detected 60, 24, and 32 different ACs in shalgams, respectively.

An important part of the ACs in shalgam comes from purple carrot, which is the main raw material used. In the current study, partially different results were obtained from the studies on carrots, the main raw material used in shalgam production. For example, Guler et al. (2015), Keser et al. (2020), Keskin et al. (2021), and Polat et al. (2022) reported that approximately 33 different ACs (terpenes, alcohols, aldehydes, ketones, acids, and esters) were detected in carrots of different colors, and the main ACs were terpenes. In the current study, because of fermentation and biochemical reactions, some new compounds, such as alcohols, were formed in shalgam.

While the ACs of all shalgam samples were generally similar, the total ACs amounts were different. The concentration of ACs in the samples varied between 5472 μg/L (Shalgam‐125 BC) and 6490 μg/L (Shalgam‐200 BC; Table 2). In previous research on the effect of different production methods on the ACs of shalgam, the ACs concentration was found to be 3162–4918 μg/L (Tanguler et al., 2017). Kirlangic et al. (2021) determined the AC concentration of shalgam at the beginning of storage as 1559–2180 μg/L. Similarly, it was reported as approximately 2230 μg/L in another study conducted by Kirlangic et al. (2021).

**TABLE 2:** Total concentrations of aroma groups of shalgam (μg/L).

Total	Shalgam‐100PC	Shalgam‐125PC	Shalgam‐150PC	Shalgam‐175PC	Shalgam‐200PC
Terpenes	(1191.48 ± 40.01)^a^	(1005.69 ± 36.72)^b^	(1048.35 ± 39.52)^b^	(1143.03 ± 37.51)^a^	(1194.04 ± 43.62)^a^
Esters	(419.51 ± 15.99)^a^	(373.72 ± 14.16)^b^	(369.87 ± 14.68)^b^	(373.18 ± 14.97)^b^	(417.02 ± 16.87)^a^
Higher alcohols	(3020.87 ± 102.16)^b^	(2949.77 ± 94.89)^c^	(3087.26 ± 79.60)^c^	(3221.08 ± 92.39)^b^	(3865.15 ± 91.16)^a^
Volatile acids	(375.29 ± 10.72)^a^	(325.01 ± 9.83)^b^	(332.19 ± 11.94)^ab^	(287.15 ± 10.32)^c^	(269.33 ± 10.72)^c^
Volatile phenols	(206.95 ± 7,70)^c^	(263.27 ± 10.92)^a^	(211.48 ± 7.13)^b^	(190.65 ± 6.65)^d^	(184.16 ± 6.35)^d^
Lactones	(99.65 ± 3.79)^b^	(99.34 ± 47.62)^b^	(101.93 ± 5.84)^b^	(143.63 ± 5.71)^a^	(92.21 ± 3.84)^b^
Norisoprenoids	(56.10 ± 2.51)^b^	(58.54 ± 2.16)^b^	(64.02 ± 2.53)^a^	(63.00 ± 2.60)^a^	(56.19 ± 2.20)^b^
Naphthalenes	(364.92 ± 6.70)^b^	(345.0 ± 8.67)^c^	(367.42 ± 15.12)^b^	(386.21 ± 13.54)^a^	(362.11 ± 13.60)^b^
Aldehydes and Ketones	(46.73 ± 1.88)^c^	(51.19 ± 2.42)^b^	(53.34 ± 2.07)^b^	(62.54 ± 2.38)^a^	49.87 ± 2.15)^b^
General total	(5781.5 ± 191.46)^b^	(5471.48 ± 227.39)^c^	(5635.86 ± 178.43)^b^	(5870.47 ± 186.07)^b^	(6490.09 ± 190.51)^a^

*Note*: The values are the means of three repetitions as μg/L; the standard deviation of all aroma groups was <5%; the differences between the amounts shown by different letters on the same line are statistically important (*p* < .05).

Abbreviations: Shalgam‐100PC, 100 g/L purple carrot addition; Shalgam‐125PC, 125 g/L purple carrot addition; Shalgam‐150PC, 150 g/L purple carrot addition; Shalgam‐175PC, 175 g/L purple carrot addition; Shalgam‐200PC, 200 g/L purple carrot addition.

### 3.2 Terpenes

It was defined in this research that terpenes were quantitatively the second group of ACs in shalgams. Similarly, Keser et al. (2020) and Keskin et al. (2021) also reported that the most important group of ACs in fresh and powdered carrots is terpenes (approximately 75%). The total amount of terpenes was observed in the Shalgam‐200PC samples reached maximum levels (1194 μg/L), followed by Shalgam‐200PC (1191 μg/L), Shalgam‐175PC (1143 μg/L), Shalgam‐150PC (1048 μg/L), and Shalgam‐125PC (1005 μg/L). Terpene compounds varied up to a certain percentage depending on the amount of carrots (*p* < .05; Table 2). As the amount of added carrots increased, the amount of total ACs increased (except for Shalgam‐100BC). However, it is interesting to note that it does not show a directly proportional change with the amount of carrots. It constitutes a certain portion (18.4–20.6%) of the total ACs in shalgams. In contrast, Ekici et al. (2021) stated that terpenes constitute half of the ACs.

Nineteen of the 26 terpenes determined and quantified in shalgams in the study carried out are terpenes determined in previous studies (Ekici et al., 2021; Tanguler et al., 2017). However, trans‐β‐farnesene (green apple), α‐zingiberene (ginger), (Z)‐γ‐bisabolene (flowery), (E)‐γ‐bisabolene (balsamic), nerol (citrus, orange), trans‐carveol (spicy), and squalene (floral) are important as they are the first time detected in shalgam. It can be said that the reason for this is the raw materials used in production, the compounds and microorganisms that pass from these raw materials to the fermentation medium, and the fermentation process.

Terpene compounds such as α‐pinene (citrus), sabinene (black pepper), β‐pinene (green, floral), β‐myrcene (spicy, floral), γ‐terpinene (sweet, citrus), terpinolene (fruity, leafy), (E)‐caryophyllene (spicy), (E)‐γ‐bisabolene (flowery), (Z)‐γ‐bisabolene (sweet–spicy–balsamic), and limonene (citrus, green) are important constituents of the carrot aroma (Keskin et al., 2021; Kreutzmann et al., 2007; Polat et al., 2022). In another study on colored carrots, it was stated that the ACs could vary according to the carrot type, and the presence of eight terpenes ((−)‐β‐pinene, β‐myrcene, d‐limonene, γ‐terpinene, α‐terpinolene, p‐cymene, (−)‐β‐caryophyllene, trans‐γ‐bisabolene) was detected in all carrot samples (Guler et al., 2015).

In the present study, α‐terpinolene (which gives oil, anise, and mint flavor) is the volatile component with the highest ratio (32.81–39.03%) of all terpenes, followed by 1‐borneol, (E)‐γ‐bisabolene, and squalene. Their amounts were determined as 368–428, 61–124, 39–142, and 70–106 μg/L, respectively (Table 1). In research investigating the effects of processing and storage methods on the ACs of shalgam, results similar to the data obtained in this study were reported (Kirlangic et al., 2021; Tanguler et al., 2017). Similarly, Ekici et al. (2021) determined the presence of terpinolene from the 10th day of shalgam fermentation and reported the importance of terpenes, a group of ACs in shalgam. Moreover, the addition of a different amount of purple carrot significantly (*p* < .05) affected the amount of relative and total terpenes.

### 3.3 Esters

Esters are compounds responsible for the floral and fruity features of many fruits, vegetables, and drinks. Esters in fermented products such as shalgam and wine come from raw materials used in production and are formed as a result of the enzymatic activity of microorganisms during fermentation (Sen, 2021; Tanguler et al., 2017). A total of seventeen ester compounds were identified in all the shalgams. Seven of them (ethyl lactate, dimethyl glutarate, ethyl octadecanoate, ethyl oleate, ethyl linoleate, ethyl vanillate, and ethyl linolenate) were not isolated and identified from shalgam in previous studies and were identified for the first time. In addition, in another study, 4 out of 34 ACs in pasteurized and unpasteurized shalgams were determined to be esters (Kirlangic et al., 2021).

The concentration of all relative ester compounds is significantly (*p* < .05) affected by the addition of the different amounts of purple carrot (Table 1). Regarding the total amounts of esters, Shalgam‐100PC and Shalgam‐200PC showed greater amounts of esters than other samples, 419.5 and 417.0 μg/L, respectively (Table 2). On the other hand, the ester contents of the Shalgam‐125PC, Shalgam‐150PC, and Shalgam‐175PC samples were similar (*p* > .05). In previous studies, the total ester content in shalgam was found to be between 105 and 173 μg/L in lower amounts, especially with the effect of the difference in the production method (Tanguler et al., 2017). In our study, the amount of ester determined in shalgams was found to be higher. This difference may depend on the production process used, the raw materials used in the manufacture, and the starter culture used.

The main ester compounds were dimethyl glutarate, ethyl hexadecanoate, ethyl lactate, isoamyl acetate, and ethyl oleate. Their amounts were determined between 80–84, 25.7–54, 34.9–43.6, 17.8–39.5 and 37–86 μg/L, respectively (Table 1). In contrast, Tanguler et al. (2017) reported that the most abundant esters in shalgams were ethyl hexadecanoate, benzyl benzoate, and isoamyl acetate, while Ekici et al. (2021) reported ethyl acetate and 3‐phenylpropyl acetate.

Ethyl lactate and isoamyl acetate were significantly higher in Shalgam‐100PC samples, although ethyl oleate was greater in Shalgam‐200PC (*p* < .05). The amounts of butyl acetate (1.13–7.75 μg/L), bornyl acetate (2.96–6.32 μg/L), and methyl salicylate (1.28–3.91 μg/L) were quite low in all samples (Table 1). It is interesting to observe that, while bornyl acetate is an ester that is detected in significant amounts (128 μg/kg) in carrots (Keser et al., 2020), it is detected in very low amounts in shalgams.

### 3.4 Higher alcohols

Many higher alcohols are synthesized during lactic acid+ethanol fermentations and contribute to the shalgam ACs. In the present study, eleven higher alcohols were described, and these are the main ACs with an amount of 52–59% in all shalgam beverages. Similarly, Ekici et al. (2021) reported that the main groups of quantitative ACs in shalgams are terpenes and alcohols. Total amounts of higher alcohols changed between 2950 μg/L (Shalgam‐125PC) and 3865 μg/L (Shalgam‐200PC), and they were the most abundant aroma group regarding their concentrations (Table 2). Tanguler et al. (2017) determined higher alcohols at lower concentrations (between 1728 and 3031 μg/L) in shalgams produced by different processes and the addition of autochthonous starter culture. While the lowest value was determined in shalgam produced by the traditional method, the highest value was reported in shalgam obtained with *Lactiplantibacillus plantarum* inoculation. In the present study, the addition of increasing amounts of purple carrots generally increased total alcohol content (exception for Shalgam‐100PC).

3‐Methyl‐1‐propanol, 1‐octanol, and benzyl alcohol are compounds that were not identified in previous studies on shalgam and were defined for the first time. Their amounts were determined between 18.8–28, 6.61–14.6 and 399–461 μg/L, respectively (Table 1). In our study, the different higher alcohols we identified may be due to fermentations, including the first (dough) fermentation, as reported by Ekici et al. (2021). On the other hand, Keskin et al. (2021) determined 3 higher alcohol components (3‐penten‐2‐ol, (z)‐3‐hexen‐1‐ol, and 2‐methyl‐2‐buten‐1‐ol) in purple carrots, while another study, 5 alcohols (2‐methyl‐1‐propanol, 3‐penten‐2‐ol, 2‐hexanol, 2‐methyl‐2‐butene‐1‐ol, and 2‐phenyl ethanol) were identified (Polat et al., 2022). Of these compounds reported in carrots, only 2‐phenyl ethanol and 2‐methyl‐1‐propanol could be detected in shalgam within the scope of this study.

The concentration of higher alcohols was highest in Shalgam‐200PC, suggesting that the amount of shalgam may affect the number of higher alcohols. Among them, isoamyl alcohol stands out significantly in terms of amount. At the same time, isoamyl alcohol had the highest concentration in all samples (1484–2206 μg/L) and the concentration was highest in Shalgam‐200PC (Table 1). This compound is one of the branched‐chain primary alcohols and is extricated as a secondary product of yeast metabolism. In many studies, isoamyl alcohol has been implicated as responsible for the cheese‐like ACs of fermented products (Celebi Uzkuc et al., 2020).

Following isoamyl alcohol, the compounds with the highest concentration in all samples were benzyl alcohol, 2‐methoxy‐phenyl‐ethanol, and 2‐phenyl ethanol. In previous research, the presence of isoamyl alcohol, 2‐methoxy‐phenyl‐ethanol, and 2‐phenyl ethanol (phenethyl alcohol) was determined, but the presence of benzyl alcohol was not in shalgam (Tanguler et al., 2017). In shalgams, benzyl alcohol with a citrus‐sweet‐like odor was determined at a concentration between 399 and 461 μg/L (Table 1) and gives shalgam a citrus‐sweet‐smelling aroma. 2‐phenyl ethanol and benzyl alcohol are compounds that give floral (roses) and citrus‐sweet aroma formation, respectively, in fermented drinks (Celebi Uzkuc et al., 2020). However, while Kirlangic et al. (2021) determined 2‐hexadecanol in shalgam, it could not be determined in our study. Thus, it is seen that different fermentation conditions and raw material amounts show different tendencies for higher alcohols. In addition, the amounts of purple carrots affected the higher alcohol concentration (*p* < .05).

### 3.5 Volatile acids

Volatile acids occur naturally in fruits, vegetables, and their juices, as well as being formed during fermentation. In fermentation, it is based on oxidation or reduction reactions of aldehydes, especially with microbial enzymes (α‐keto acid dehydrogenase) synthesized by lactic acid bacteria (Tanguler et al., 2017). The total amount of volatile acids ranged from 325 to 375 μg/L in Shalgam‐100PC, Shalgam‐125PC, and Shalgam‐150PC samples; on the other hand, this value was 269 and 287 μg/L in Shalgam‐175 BC and Shalgam‐200PC, respectively (Table 2). Kirlangic et al. (2021) reported the total amount of volatile acid in unpasteurized shalgam as 89.5 μg/L. In previous research, these values were between 69.3 and 163 μg/L in all samples (Tanguler et al., 2017) and were found to be lower than the values obtained in our study. A decrease in total volatile acid amount was observed with increasing purple carrot addition, and it was found to be statistically significant (*p* < .05). It constitutes 4.15–6.49% of all ACs.

In the purple carrot studies, a volatile acid, hexanoic acid, was found and its amount was expressed as 133 and 141.7 μg/kg. They reported that acetic acid could not be determined in fresh carrots (Keser et al., 2020; Keskin et al., 2021). In contrast to the other two studies, Polat et al. (2022) stated the presence of four volatile acids (acetic acid, hexanoic acid, octanoic acid, and nonanoic acid) in purple carrots, with this volatile acid comprising only 1.23% of all ACs. Therefore, it can be said that some of these compounds determined in shalgam originate from purple carrot.

Ten volatile acids were determined in all shalgam samples. In all samples, the main volatile acid compounds were heptanoic acid (ranging from 117.8 to 153.1 μg/L) and hexanoic acid (ranging from 63.6 to 92.6 μg/L), respectively (*p* < .05; Table 1). Both compounds are characterized as odorous and plastic‐like (Schreiner et al., 2018). Other ones are decanoic, acetic, butanoic, isovaleric, 2‐methyl‐butanoic, pentanoic, octanoic, and dodecanoic acid. Kirlangic et al. (2021) identified 2 acids (acetic acid and nonanoic acid) in shalgam samples. Similarly, in a previous study, five different ACs were found: butanoic acid, isovaleric acid, pentanoic acid, hexanoic acid, and heptanoic acid (Tanguler et al., 2017). However, in our current study, 2‐methyl‐butanoic acid, octanoic acid, decanoic acid, and dodecanoic acid were detected for the first time in shalgam. Therefore, as stated above, it can be said that the volatile acids determined other than octanoic acid originate from fermentation.

### 3.6 Volatile phenols

Shalgam‐100PC and Shalgam‐150PC samples contained 206.95 and 211.48 μg/L of volatile phenols, respectively. This Shalgam‐125PC (263.3 μg/L) sample contained more volatile phenol compounds in quantity than the others, and increased purple carrot content generally resulted in a decrease in volatile phenol content (*p* < .05; Table 2). Polat et al. (2022) found only one phenolic volatile (2,6‐bis(1,1‐dimethyl ethyl)‐4‐methyl phenol) compound in fresh purple carrot pomace samples. On the other hand, Keskin et al. (2021) reported 3,5‐di‐tert‐butyl‐phenol as a volatile phenolic in addition to the study of Polat et al. (2022) in purple carrots. In the present study, nine volatile phenols were identified and quantified in the shalgam. 4‐Methyl‐2,6‐di‐tert‐butyl phenol, eugenol, and guaiacol were quantitatively important main volatile phenols. Their amounts were determined between 51.4–89 μg/L, 31.8–56 μg/L, and 22.6–58.6 μg/L, respectively (Table 1). 4‐Methyl‐2,6‐di‐tert‐butyl‐phenol has been associated with burnt‐plastic odors, while eugenol provides a clove‐like fragrance. Eugenol is an important aroma‐active substance in some fruits and has a low detection threshold (6 μg/L) in aqueous media (Tanguler et al., 2017). Besides guaiacol, 2‐methoxy‐4‐vinyl‐phenol and cis‐isoeugenol are volatile phenols that have not been identified so far in shalgams and are reported for the first time in a study. In a previous study, in addition to eugenol, p‐vinyl guaiacol was the main volatile phenol (Tanguler et al., 2017). In another study, 9 volatile phenols, such as 2,4‐di‐tert butyl‐phenol, were determined in shalgams stored at 4 and 30°C for 2 months (Kirlangic et al., 2021).

### 3.7 Lactones

Considering the total concentrations of lactones, Shalgam‐175PC samples resulted in a greater amount of lactones (143.63 μg/L) in all samples. On the other hand, the difference between the other samples was found to be statistically insignificant (*p* > .05). A total of 6 lactone compounds were identified and quantified in all samples. γ‐Nonalactone, with a sweet, coconut‐like aroma, was the main lactone, and its amount was higher in Shalgam‐175 BC (73.1 μg/L) than the others. Moreover, this lactone nearly accounted for 43–65% of total lactones in all samples in the present study (Table 1). Another important lactone in terms of amount, γ‐dodecalactone, has a peach‐like aroma, and its amounts were found between 7.1 and 37.7 μg/L in shalgam samples (Schreiner et al., 2018). On the other hand, in this study, five lactones (γ‐butyrolactone, γ‐hexalactone, δ‐dodecalactone, and γ‐nonalactone massoilactone) were detected, similar to previous research (Tanguler et al., 2017). However, dihydro‐4‐hydoxy‐2(3H)‐furanone was detected for the first time in shalgams.

### 3.8 Naphthalenes

Naphthalene can occur during the dehydration and cyclization of hydrocarbon compounds under a certain temperature, or it can be formed by the degradation of fruits/vegetables by some microorganisms present in the medium. Naphthalenes have been reported to be responsible for the phenolic odor, which is generally accepted as unpleasant (Li et al., 2021; Tanguler et al., 2017).

Considering the total naphthalene concentrations, the lowest value was found in the Shalgam‐125PC sample as 345 μg/L, while the highest value was found in Shalgam‐175PC (386 μg/L; *p* < .05). Concerning naphthalene compounds, naphthalene, 1‐methyl‐naphthalene, 2‐methyl‐naphthalene, 1‐ethyl‐naphthalene, and 2‐ethyl‐naphthalene were determined in the shalgam samples. Of these compounds, 1‐methyl‐naphtalene and 1‐ethyl‐naphtalene were determined in shalgam for the first time in the present study. Naphthalene was found the most (165–201 μg/L) in all samples, followed by 2‐ethyl‐naphthalene (137–150 μg/L; Table 1). In a previous study (Tanguler et al., 2017) on the ACs of shalgam with different production methods, three naphthalenes (naphthalene, 2‐methyl‐naphthalene, and 2‐ethyl‐naphthalene) were found, and their values are consistent with the values determined in the current study. On the other hand, components such as naphthalene, 2‐methyl‐naphthalene, 1‐methylnaphthalene, and 1,6‐dimethyl‐naphthalene have been detected in foxtail millet varieties (Li et al., 2021).

### 3.9 Other compounds

Norisoprenoids can form by degrading aromatic carotenoids like beta‐carotene and neoxanthin directly, or they can be stored as glycoconjugates that can subsequently release their volatile aglycone during fermentation through enzymatic and acid hydrolysis processes. These compounds, which have low detection thresholds, are particularly characterized by imparting floral and woody odors to fermented foods/drinks (Mendes‐Pinto, 2009). 3‐Hydroxy‐β‐damascenone, a norisoprenoid previously identified in shalgam, has a very low detection threshold. This compound has a significant effect on the aroma compounds of food and drinks. In a previous study, 3‐hydroxy‐β‐damascenone as a norisoprenoid was defined, and its amounts were reported to be between 37.5 and 69.2 μg/L (Tanguler et al., 2017). Similarly, in our study, it was determined between 47.5 and 55.1 μg/L (Table 1). In contrast, 3‐oxo‐α‐ionol was detected for the first time in shalgams.

In the present study, the total amount of aldehydes and ketones changed from 46.7 (Shalgam‐100PC) to 62.5 μg/L (Shalgam‐175PC; *p* < .05). Similar to norisoprenoids, aldehydes and ketones were found to be the least dominant compounds in number and amount compared to other aroma compounds. 4‐hydroxy‐4‐methyl‐2‐pentanone and heptanal were detected in trace quantities (45.5–61.1 and 1.14–1.48 μg/L, respectively). Statistical differences were observed with a slight variation in the 4‐hydroxy‐4‐methyl‐2‐pentanone and heptanal concentrations of the samples with varying amounts of purple carrots (*p* < .05; Tables 1 and 2).

Guler et al. (2015) detected 3 aldehydes, acetaldehyde, hexanal, and octanal, in all carrot cultivars. In another study, 3‐hydroxy‐3‐methyl‐2‐butanone, 3‐hydroxy‐2‐butanone, 1‐hydroxy‐2‐propanone, furfural, and nonanal were identified as carbonyl compounds in raw, colored carrots, and their total concentration was determined to be 569 μg/kg (Keskin et al., 2021). Keser et al. (2020) reported that the dominant aldehydes in carrots are hexanal, heptanal, and octanal. Similarly, Polat et al. (2022) found furfural in processed carrots in addition to hexanal, heptanal, nonanal, and 3‐hydroxy‐2‐butanone determined in raw carrots. In research, the presence of 3 aldehydes and 4 ketone compounds in shalgam was reported (Kirlangic et al., 2021). Also, other studies reported the presence of hexanal and heptanal as aldehydes and 4‐nonanone, 4‐hydroxy‐4‐methyl‐2‐pentanone, and 2‐heptanone as ketones (Ekici et al., 2021; Tanguler et al., 2017). In this study, aldehydes and ketones were determined, and their amounts are consistent with previous studies.

**FIGURE 2:**
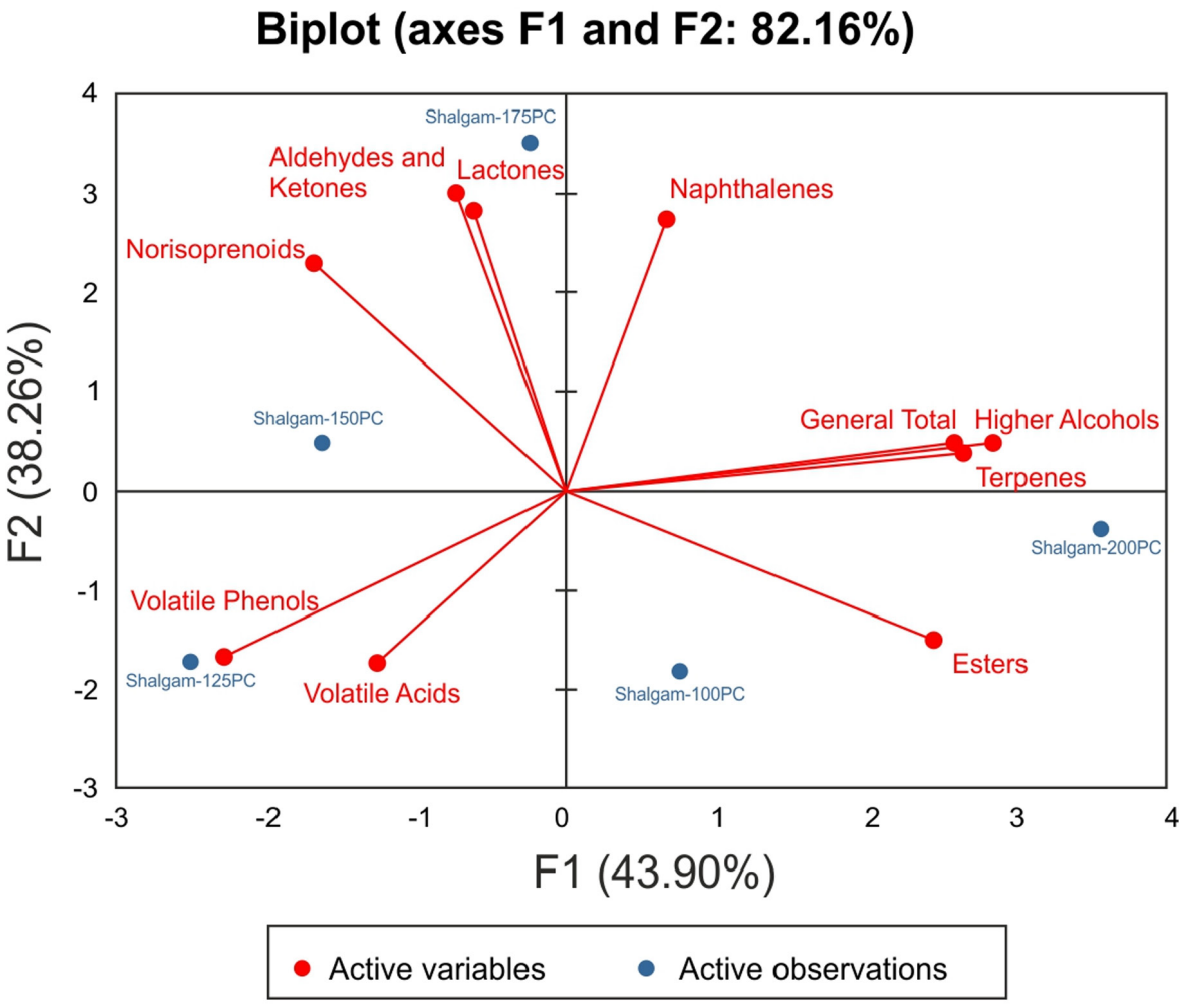
Principal component analysis (PCA) biplot of shalgam samples. Shalgam‐100PC, 100 g/L purple carrot addition; Shalgam‐125PC, 125 g/L purple carrot addition; Shalgam‐150PC, 150 g/L purple carrot addition; Shalgam‐175PC, 175 g/L purple carrot addition; Shalgam‐200PC, 200 g/L purple carrot addition.

Principal component analysis (PCA) was applied to construct a model to classify shalgam samples according to aroma groups. As seen in the biplots (Figure 2), the PCA model, which consists of two main components, explains 82.16% of the total variance (F1: 43.90%, F2: 38.26%). According to the biplot of the aroma groups, Shalgam‐125PC, Shalgam‐150PC, and Shalgam‐175PC samples are on the left, and Shalgam‐100PC and Shalgam‐200PC samples are on the right. It has been determined that the correlation between the location of the shalgam samples and the aroma groups is important in the coordinate system. Shalgam‐150PC and Shalgam‐175PC samples showed a dominant character in terms of norisoprenoid, aldehyde, and ketone compounds. Similarly, higher alcohol compounds, terpenes, and esters were found to be dominant in the Shalgam‐200PC sample. The Shalgam‐125PC sample, on the other hand, showed a more dominant character in terms of volatile phenol and volatile acid compounds compared to the other samples. Therefore, it is seen that the effect of adding different amounts of carrots to the ACs in shalgam is significant.

## 4 CONCLUSION

One of the most important results obtained in this study is that the use of different amounts of purple carrots during shalgam production significantly affects ACs both quantitatively and qualitatively. Furthermore, the maximum total amount of ACs was produced with the addition of 200 g/L purple carrot. Moreover, twenty‐eight ACs were detected for the first time in shalgam, including 7 terpenes, 7 esters, 3 alcohols, 4 volatile acids, 3 volatile phenols, 1 lactone, 1 norisoprenoid, and 2 naphthalenes. In this respect, the new findings obtained within the scope of this study have the potential to benefit the shalgam‐producing industry and researchers who strive to improve the nutritional value, processing, and marketing of shalgam. In particular, it can give producers an idea of how much carrot to use in production in terms of aroma, which affects the appeal of consumers. However, there is a need to conduct various studies, especially on the extent to which the fermentation process will affect the shalgam beverage ACs affected by the addition of purple carrots.

## AUTHOR CONTRIBUTIONS

**Hasan Tanguler:** Conceptualization (equal); data curation (equal); formal analysis (equal); investigation (equal); methodology (equal); project administration (lead); resources (equal); writing – review and editing (equal). **Kemal Sen:** Conceptualization (equal); data curation (equal); formal analysis (equal); investigation (equal); methodology (equal); resources (equal); validation (lead); writing – review and editing (equal). **Selin Ozge Dinc:** Investigation (equal); visualization (supporting); writing – original draft (lead); writing – review and editing (equal).

## CONFLICT OF INTEREST STATEMENT

The authors declare that they have no known financial interests or personal relationships that could influence the work reported in this article.

## Data Availability

The data used to support the findings of this study can be made available by the corresponding author upon request.

## References

[fsn34164-bib-0001] Alabran, D. M. , & Mabrouk, A. F. (1973). Carrot flavour. Sugars and free nitrogenous compounds in fresh carrots. Journal of Agricultural and Food Chemistry, 21, 205–208.

[fsn34164-bib-0002] Angerosa, F. (2002). Influence of volatile compounds on virgin olive oil quality evaluated by analytical approaches and sensor panels. European Journal of Lipid Science and Technology, 104, 639–660.

[fsn34164-bib-0003] Celebi Uzkuc, N. M. , Şişli, B. , Ay, M. , Togay, S. O. , Karagül Yüceer, Y. , Bayhan, A. , & Kırca Tokluca, A. (2020). Effects of spontaneous fermentation on Karalahna and cabernet sauvignon young red wines: Volatile compounds, sensory profiles and identification of autochthonous yeasts. European Food Research and Technology, 246(1), 81–92. 10.1007/s00217-019-03395-w

[fsn34164-bib-0004] Ekici, H. , Kadiroglu, P. , & Ilgaz, C. (2021). Next‐generation sequencing of shalgam flavor influencing microflora. Journal of Food Processing and Preservation, 46(6), e15982. 10.1111/jfpp.15982

[fsn34164-bib-0005] Ekinci, F. Y. , Baser, G. M. , Ozcan, E. , Ustundağ, O. G. , Korachi, M. , Sofu, A. , Blumberg, J. B. , & Chen, C. Y. O. (2016). Characterization of chemical, biological, and antiproliferative properties of fermented black carrot juice, shalgam. European Food Research and Technology, 242(8), 1355–1368. 10.1007/s00217-016-2639-7

[fsn34164-bib-0006] Erten, H. , & Tanguler, H. (2012). Shalgam (Şalgam). In Y. H. Hui (Ed.), Handbook of plant based fermented food and beverage technology (pp. 657–664). CRC Press.

[fsn34164-bib-0007] Erten, H. , Tanguler, H. , & Canbaş, A. (2008). A traditional Turkish lactic acid fermented beverage: Shalgam (Salgam). Food Reviews International, 24(3), 352–359. 10.1080/87559120802089324

[fsn34164-bib-0008] Guler, Z. , Karaca, F. , & Yetisir, H. (2015). Identification of volatile organic compounds (VOCs) in different colour carrot (*Daucus carota* L.) cultivars using static headspace/gas chromatography/mass spectrometry. Cogent Food and Agriculture, 1(1), 1117275. 10.1080/23311932.2015.1117275

[fsn34164-bib-0009] Kesen, S. (2020). Characterization of aroma and aroma‐active compounds of Turkish turmeric (*Curcuma longa*) extract. Journal of Raw Materials to Processed Foods, 1(1), 13–21.

[fsn34164-bib-0010] Keser, D. , Guclu, G. , Kelebek, H. , Keskin, M. , Soysal, Y. , Sekerli, Y. E. , Arslan, A. , & Selli, S. (2020). Characterization of aroma and phenolic composition of carrot (*Daucus carota ‘Nantes’*) powders obtained from intermittent microwave drying using GC–MS and LC‐MS/MS. Food and Bioproducts Processing, 119, 350–359. 10.1016/j.fbp.2019.11.016

[fsn34164-bib-0011] Keskin, M. , Guclu, G. , Sekerli, Y. E. , Soysal, Y. , Selli, S. , & Kelebek, H. (2021). Comparative assessment of volatile and phenolic profiles of fresh black carrot (*Daucus carota* L.) and powders prepared by three drying methods. Scientia Horticulturae, 287, 110256. 10.1016/j.scienta.2021.110256

[fsn34164-bib-0012] Kirlangic, O. , Ilgaz, C. , & Kadiroğlu, P. (2021). Influence of pasteurization and storage conditions on microbiological quality and aroma profiles of shalgam. Food Bioscience, 44, 101350. 10.1016/j.fbio.2021.101350

[fsn34164-bib-0013] Kreutzmann, S. , Thybo, A. K. , & Bredie, W. L. P. (2007). Training of a sensory panel and profiling of winter hardy and coloured carrot genotypes. Food Quality and Preference, 18(3), 482–489. 10.1016/j.foodqual.2006.05.009

[fsn34164-bib-0014] Li, S. , Zhao, W. , Liu, S. , Li, P. , Zhang, A. , Zhang, J. , Wang, Y. , Liu, Y. , & Liu, J. (2021). Characterization of nutritional properties and aroma compounds in different colored kernel varieties of foxtail millet (*Setaria italica*). Journal of Cereal Science, 100, 103248. 10.1016/j.jcs.2021.103248

[fsn34164-bib-0015] McFeeters, R. F. (2004). Fermentation microorganism and flavour changes in fermented foods. Journal of Food Science, 69, 35–37.

[fsn34164-bib-0016] Mendes‐Pinto, M. M. (2009). Carotenoid breakdown products the norisoprenoids in wine aroma. Archives of Biochemistry and Biophysics, 483(2), 236–245. 10.1016/j.abb.2009.01.008 19320050

[fsn34164-bib-0017] Polat, S. , Guclu, G. , Kelebek, H. , Keskin, M. , & Selli, S. (2022). Comparative elucidation of colour, volatile and phenolic profiles of black carrot (*Daucus carota* L.) pomace and powders prepared by five different drying methods. Food Chemistry, 369, 130941. 10.1016/j.foodchem.2021.130941 34479009

[fsn34164-bib-0018] Schreiner, L. , Bauer, P. , & Buettner, A. (2018). Resolving the smell of wood‐identification of odour‐active compounds in scots pine (*Pinus sylvestris* L.). Scientific Reports, 8(1), 8294. 10.1038/s41598-018-26626-8 29844440 PMC5974339

[fsn34164-bib-0019] Sen, K. (2021). The influence of different commercial yeasts on aroma compounds of rosé wine produced from cv. Öküzgözü grape. Journal of Food Processing and Preservation, 45(7), e15610. 10.1111/jfpp.15610

[fsn34164-bib-0020] Sonmezdag, A. S. , Kelebek, H. , & Selli, S. (2018). Pistachio oil (*Pistacia vera L*. cv. Uzun): Characterization of key odorants in a representative aromatic extract by GC‐MS‐olfactometry and phenolic profile by LC‐ ESI‐ MS/MS. Food Chemistry, 240, 24–31. 10.1016/j.foodchem.2017.07.086 28946268

[fsn34164-bib-0021] Tanguler, H. , & Erten, H. (2012). Chemical and microbiological characteristics of shalgam (salgam); a traditional Turkish lactic acid fermented beverage. Journal of Food Quality, 35, 298–306. 10.1111/j.1745-4557.2012.00447.x PMC5495728 28720958

[fsn34164-bib-0022] Tanguler, H. , Gunes, G. , & Erten, H. (2014). Influence of addition of different amounts of black carrot (*Daucus carota*) on shalgam quality. Journal of Food, Agriculture and Environment, 12(2), 60–65.

[fsn34164-bib-0023] Tanguler, H. , Selli, S. , Sen, K. , Cabaroglu, T. , & Erten, H. (2017). Aroma composition of shalgam: A traditional Turkish lactic acid fermented beverage. Journal of Food Science and Technology‐Mysore, 54(7), 2011–2019. 10.1007/s13197-017-2637-1 PMC5495728 28720958

[fsn34164-bib-0024] Tanriseven, D. , Kadiroglu, P. , Selli, S. , & Kelebek, H. (2020). LC‐DAD‐ESI‐MS/MS‐assisted elucidation of the phenolic compounds in shalgams: Comparison of traditional and direct methods. Food Chemistry, 305, 125505. 31606693 10.1016/j.foodchem.2019.125505

[fsn34164-bib-0025] TSE . (2003). TS 11149 standard of Shalgam beverage. Turkish Standards Institute.

[fsn34164-bib-0026] Türk, G. , & Şen, K. (2021). Changes of various quality characteristics and aroma compounds of astragalus honey obtained from different altitudes of Adana‐Turkey. Journal of Food Processing and Preservation, 45(10), e15852. 10.1111/jfpp.15852

